# Characterization of Tonsil Microbiota and Their Effect on Adenovirus Reactivation in Tonsillectomy Samples

**DOI:** 10.1128/Spectrum.01246-21

**Published:** 2021-10-20

**Authors:** Lingling Wang, Dongge Xu, Qun Huang, Guang Yang, Mengyu Zhang, Jingai Bi, Jinjun Shan, Erguang Li, Susu He

**Affiliations:** a State Key Laboratory of Pharmaceutical Biotechnology, Medical School, Nanjing University, Nanjing, China; b Jiangsu Key Laboratory of Molecular Medicine, Medical School, Nanjing University, Nanjing, China; c Yancheng Medical Research Center, Medical School, Nanjing University, Yancheng, China; d Nanjing Children's Hospital, Nanjing Medical University, Nanjing, China; e Institute of Medical Virology, Nanjing Drum Tower Hospital, Medical School, Nanjing University, Nanjing, China; f The First Medical College, Nanjing University of Chinese Medicine, Nanjing, China; g Shenzhen Research Institute of Nanjing University, Shenzhen, China; Lerner Research Institute

**Keywords:** adenovirus, latency, reactivation, microbiota, SCFA, adenovirus

## Abstract

The adenoviral DNA is prevalent in adenotonsillectomy specimens from pediatric patients, though the virus seems to be in latent state. The tonsils are at the forefront of airway entry point and are the first line of defense against airway viral and bacterial infections. We hypothesized that tonsil microbiota plays a role in human adenovirus (HAdV) latency and reactivation. In this study, we surveyed the presence of HAdV in tonsillectomy samples from 81 patients and found that HAdV DNA was in 85.2% of the tonsil samples. We then determined the microbiota of the samples. Taxonomic profiling showed that *Proteobacteria*, *Firmicutes*, *Fusobacteriota*, and *Bacteroidota* accounted for approximately 70% of the total phyla in tonsil samples. A correlation analysis showed that the HAdV-positive samples had significantly higher abundance of *Neisseria* and *Bifidobacterium* and lower abundance of Streptococcus, *Ochrobactrum*, and *Lactobacillus* than that of the HAdV-negative samples. Culture-based isolation followed by 16S rRNA sequencing identified Staphylococcus aureus, Streptococcus pneumoniae, Veillonella, *Prevotella,* Capnocytophaga sputigena, Pseudomonas aeruginosa, *Neisseria*, and Moraxella catarrhalis from the samples. Gas chromatography-mass spectrometry (GC-MS) profiling of short-chain fatty acids in bacterial cultures of minced tonsillectomy tissues or representative isolates showed the cultures contained various amounts of short-chain fatty acids (SCFAs). Treatment of isolated tonsil lymphocytes with bacterial lipopolysaccharide (LPS) or with SCFAs promoted HAdV reactivation. The compounds also promoted HAdV reactivation in a xenograft model with implanted tonsil fragments. This study shows a potential interplay between tonsil microbiota and HAdV reactivation that may lead to recurrent virus infection of respiratory tract disease.

**IMPORTANCE** Human adenovirus infection is common among pediatric patients and can be life-threatening among organ transplant recipients. Adenovirus is transmitted by close contact, but it is believed that a majority of invasive events appear to arise from viral reactivation. The human tonsil is a reservoir for virus latency and has a high prevalence of latently infected adenovirus. Also, tonsils are located at the gateway of the respiratory tracts and are commonly exposed to bacterial pathogens. Here, we uncovered adenoviral DNA-positive and -negative samples that appeared to harbor distinct distribution patterns of microorganisms. SCFAs, primary metabolites of microbiota on tonsils, could induce the adenovirus reactivation in tonsil lymphocytes, resulting in adenovirus replication and production of infectious virions. The study suggests that viral-bacterial interaction plays a role in virus reactivation from latency and could be a contributing factor for recurrent viral infection in pediatric patients.

## INTRODUCTION

Respiratory tract infection (RTI) by viruses, bacteria, and even fungi is common among people of all age groups (1, 2). Respiratory infections such as influenza often have strong seasonal patterns; others, like adenovirus infection, occur throughout the year. Human adenoviruses (HAdVs) are common pathogens of humans and animals. More than 50 distinct serotypes of HAdV have been identified (3, 4). HAdV infections cause a range of clinical manifestations in immunocompetent individuals, most commonly acute respiratory illness (ARI), gastroenteritis, and conjunctivitis (3, 5). HAdV infections of the upper respiratory tract are common yet subclinical, including common cold symptoms, pharyngitis, tonsillitis, otitis media, and pharyngoconjunctival fever (6, 7). Life-threatening disseminated infection and lower respiratory infection, like severe pneumonia and encephalitis, occur occasionally, especially among young infants and immunocompromised patients (8, 9). Despite recent progress in the diagnostic risk assessment of HAdV infections in immunocompromised patients, clinical complications mediated by these viruses continue contributing to significant morbidity and mortality, particularly in hematopoietic allogeneic stem cell transplant (HSCT) and solid organ transplant recipients (6, 8, 10).

HAdVs are double-stranded, nonenveloped DNA viruses of the *Adenoviridae* family and *Mastadenovirus* genus. The HAdV was first isolated from removed adenoids by Rowe in 1953 (11). Shortly after the isolation of HAdV, Evans and colleagues reported latent adenovirus infections of the human respiratory tract (12). Evans defined adenovirus “latent infection” as a situation in which the virus persists in the host cells without producing obvious symptoms. Unlike the herpesviruses such as herpes simplex virus (HSV), varicella-zoster virus (VZV), and Epstein-Barr virus (EBV) that have well-defined life cycles of lysogenic infection and latency, the latency or persistency of HAdV infection is less well characterized, although HAdV reactivation can be life-threatening to certain patients (13).

It has been known for a long time that viral infections after HSCT are frequently caused by the endogenous reactivation of persistent pathogens such as human cytomegalovirus (HCMV), EBV, and HAdV (3, 14). Although exogenous infections occur, accumulating evidence strongly implicates viral reactivation to the high incidence of HAdV infections in HSCT and solid organ transplant recipients (14–16). In pediatric patients, HAdV DNA was detected in approximately 80% of tonsillectomy samples (17, 18). A recent study reported HAdV DNA in 80% of the 35 adenoid tissue samples, more than any other viruses like HHV-7 at 51.4% and EBV at 42.9% (19). Although HAdV DNA is prevalent in adenoids and tonsils, HAdV transcripts have rarely been detected in uncultured lymphocytes (17, 20–22), suggesting that the virus is likely in latent or persistent status. People with chronic adenotonsillar diseases have persistent nonproductive HAdV infection (23, 24), although the mechanism by which HAdV reactivation is regulated has been less investigated.

The adenoid tonsil and the tubal tonsils, palatine tonsils, and lingual tonsils are collectively called tonsils. The tonsils serve as the first line of defense of the immune system against ingested or inhaled foreign pathogens. The mucous surfaces of the tonsils and the airway tracts are colonized by a wide range of bacteria, though the majority of them belong to normal flora. Bacteria such as group A beta-hemolytic *Streptococci* and Pseudomonas are among the common pathogens causing strep throat and tonsillitis. Also, the tonsils are known as a reservoir for latently infected viruses such as EBV and likely HAdV and can be a source for recurrent infections. We and others have reported that HAdV DNA was prevalent in the tonsillectomy samples. The virus seems to be in a latent state since viral gene expression is rarely detected in the samples without stimulation (18, 20). HDAC inhibitors and reagents promoting protein kinase C (PKC) signaling cause HAdV reactivation (18, 25), while bacterially produced short-chain fatty acids (SCFAs) can increase histone acetylation (26, 27). Since bacterial lipopolysaccharide (LPS) and SCFA are known to promote histone acetylation (27–29), we questioned whether there was an interplay between bacterial colonization and HAdV reactivation through bacterial metabolites.

In this study, we surveyed tonsillectomy samples from 81 patients for the presence of HAdV and investigated the microbiota on the samples. We found that the samples, mainly in T lymphocytes, contained HAdV DNA. A correlation analysis showed a distinct difference in the surface microbiota among HAdV-positive and HAdV-negative tonsillar samples. We isolated different microbes and showed that bacterial metabolites like SCFAs caused HAdV reactivation. Human tonsils serve as a reservoir for virus latency. The study highlights the underlying interplay between bacterial and viral infections on respiratory tract diseases and potentially links microbial activity to recurrent virus infection.

## RESULTS

### Detection of viral DNA in tonsillectomy samples.

Tonsil samples from 81 patients were collected from patients undergoing tonsillectomy procedures at Nanjing Children’s Hospital due to diagnosed tonsillar hypertrophy or chronic/recurrent tonsillitis. The age of the patients was between 1.1 and 12.9 years old. The patient information and blood test results, including counts of lymphocytes, neutrophils, platelets, and hemoglobin and C-reactive protein (CRP) contents, are listed in Tables 1 and 2. All parameters were within normal ranges and were not significantly different from those of healthy donors. This is consistent with the observation that none of these patients had infective symptoms on the day of surgery.

**TABLE 1:** Demographic characteristics of the participants and HAdV DNA status

Characteristic	Data for:	
	HAdV-positive patients (*n* = 69)	HAdV-negative patients (*n* = 12)
Symptom (no. of patients)		
Recurrent tonsillitis	5	2
Hypertrophic tonsils	64	10
Gender (no. [%])		
Male	40 (49.4)	7 (8.6)
Female	29 (35.8)	5 (6.2）
Age (mean ± SD [yrs])	5.69 ± 2.08	5.02 ± 1.46
Age group (no. [%])		
0 to ≤3 yrs	3 (3.7)	1 (1.2)
3 to ≤6 yrs	40 (49.4)	8 (9.9)
6 to ≤9 yrs	21 (25.9)	3 (3.7)
>9 yrs	5 (6.2)	0 (0)
		
Blood test result		
WBC^*a*^ (mean ± SD [10^9^ cells/liter])	8.79 ± 5.37	8.45 ± 3.89
Leukopenia (mean ± SD [<4,000/μl])	56.34 ± 14.58	45.63 ± 9.69
Lymphocytes (mean ± SD, %)	34.78 ± 13.57	40.45 ± 11.54
Neutrophils (mean ± SD, %)	53.45 ± 10.43	44.21 ± 8.46
Platelets (mean ± SD [10^9^ cells/liter])	268.51 ± 88.97	287.21 ± 76.42
Hemoglobin (mean ± SD [g/liter])	123.71 ± 13.24	125.43 ± 8.45
CRP > 8 mg/liter (no. [%])	15 (18.5)	1 (1.2)

a WBC, white blood cell.

**TABLE 2:** Demographic characteristics of the participants and EBV DNA status

Characteristic	Data for:	
	EBV-positive patients (*n* = 48)	EBV-negative patients (*n* = 33)
Symptom (no. of patients)		
Recurrent tonsillitis	4	3
Hypertrophic tonsils	44	30
Gender (no. [%])		
Male	20 (24.7)	20 (24.7)
Female	28 (34.6)	13 (16.0）
Age (mean ± SD [yrs])	6.31 ± 1.34	5.74 ± 1.89
Age group (no. [%])		
0 to ≤3 yrs	2 (2.5)	2 (2.5)
3 to ≤6 yrs	25 (30.9)	23 (28.3)
6 to ≤9 yrs	19 (23.4)	5 (6.2)
>9 yrs	2 (2.5)	3 (3.7)
		
Blood test result		
WBC (mean ± SD [10^9^ cells/liter])	8.15 ± 3.26	9.16 ± 2.80
Leukopenia (mean ± SD [<4,000/μl])	55.82 ± 13.74	44.62 ± 8.72
Lymphocytes (mean ± SD, %)	33.31 ± 11.73	43.67 ± 10.22
Neutrophils (mean ± SD, %)	55.73 ± 12.68	42.46 ± 7.93
Platelets (mean ± SD [10^9^ cells/liter])	287.12 ± 67.65	278 ± 74.13
Hemoglobin (mean ± SD [g/liter])	125 ± 8.14	126 ± 9.15
CRP > 8 mg/liter (no. [%])	13 (16.0)	3 (3.7)

We assessed the prevalence of HAdV, EBV, and HCMV in human palatine tonsils by PCR. HAdV DNA was detected in 69 of the 81 patients (85.2%) (Table 1), while EBV DNA was detected in 48 patients (59.2%) (Table 2). Only 2 patients (2.5%) had a detectable amount of HCMV in the tonsil samples. Of all the patients, 40 (49.4%) patients had both HAdV and EBV. No patients had a coinfection of HCMV with the other two viruses. We also performed reverse transcriptase PCR (RT-PCR) studies to detect HAdV gene expression. Only 2 samples had detectable viral E1A mRNA, representing a rate of approximately 2.5%. However, lymphocytes isolated from the samples failed to produce infectious virions detected on the susceptible HEp-2 cells.

**FIG 1:**
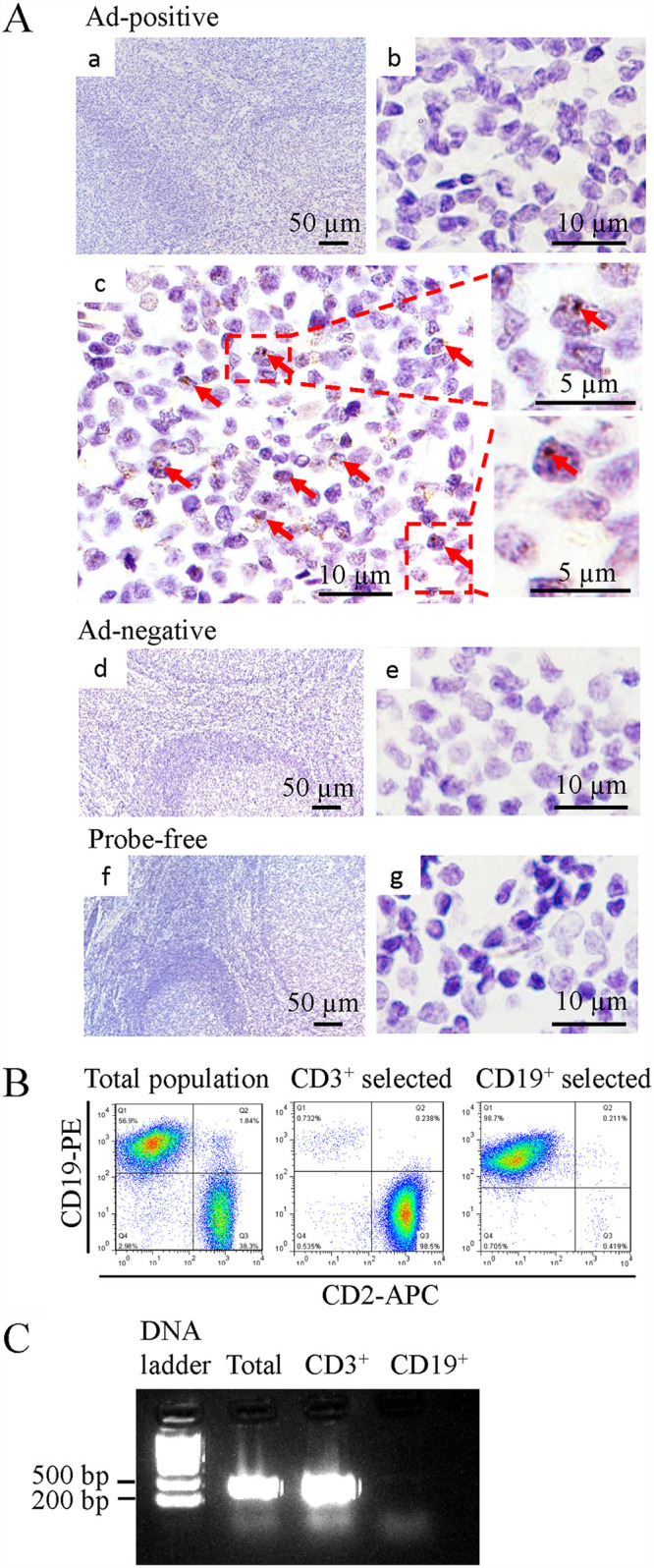
Distribution of HAdV in the tonsil. (A) *In situ* hybridization. (a) HAdV-positive sample, low magnification; (b) high magnification, lymphoid follicles, no HAdV DNA signal; (c) high magnification, interfollicular area, HAdV DNA in brown and indicated by arrows; (d and e) HAdV-negative tissue; (f and g) HAdV-positive tissue without probe. Scale bars are identified in the panels. (B) Lymphocytes typing for HAdV latent infection. Isolation of CD3^+^ T and CD19^+^ B cells by magnetic bead sorting. (C) Detection of HAdV DNA in isolated CD3^+^ T and CD19^+^ B cells by PCR using hexon primers.

We performed fluorescent *in situ* hybridization (FISH) to confirm the presence of HAdV DNA in paraffin-embedded tonsil tissue sections with an HAdV probe. Consistent with the results from the PCR study, no specific staining was visible in HAdV DNA-negative samples. In HAdV DNA-positive samples, the staining was spotted mainly in the interfollicular area (Fig. 1A).

The presence of HAdV in B or T lymphocytes was confirmed by PCR studies using antibody-purified cells. In this regard, the lymphocytes were separated into B and T lymphocytes using antibody-coated magnetic beads. The purity of the cell populations was determined by fluorescence-activated cell sorter (FACS) analysis to be 95% or better for lymphocytes (Fig. 1B). We found that adenoviral DNA was detected in the subset of CD3^+^ T cell lymphocytes using a pair of primers that anneal to the hexon gene of species B, C, D, and E of HAdV (Fig. 1C), whereas EBV DNA was detected in the B cell population (data not shown).

### 16S rRNA analysis.

**FIG 2:**
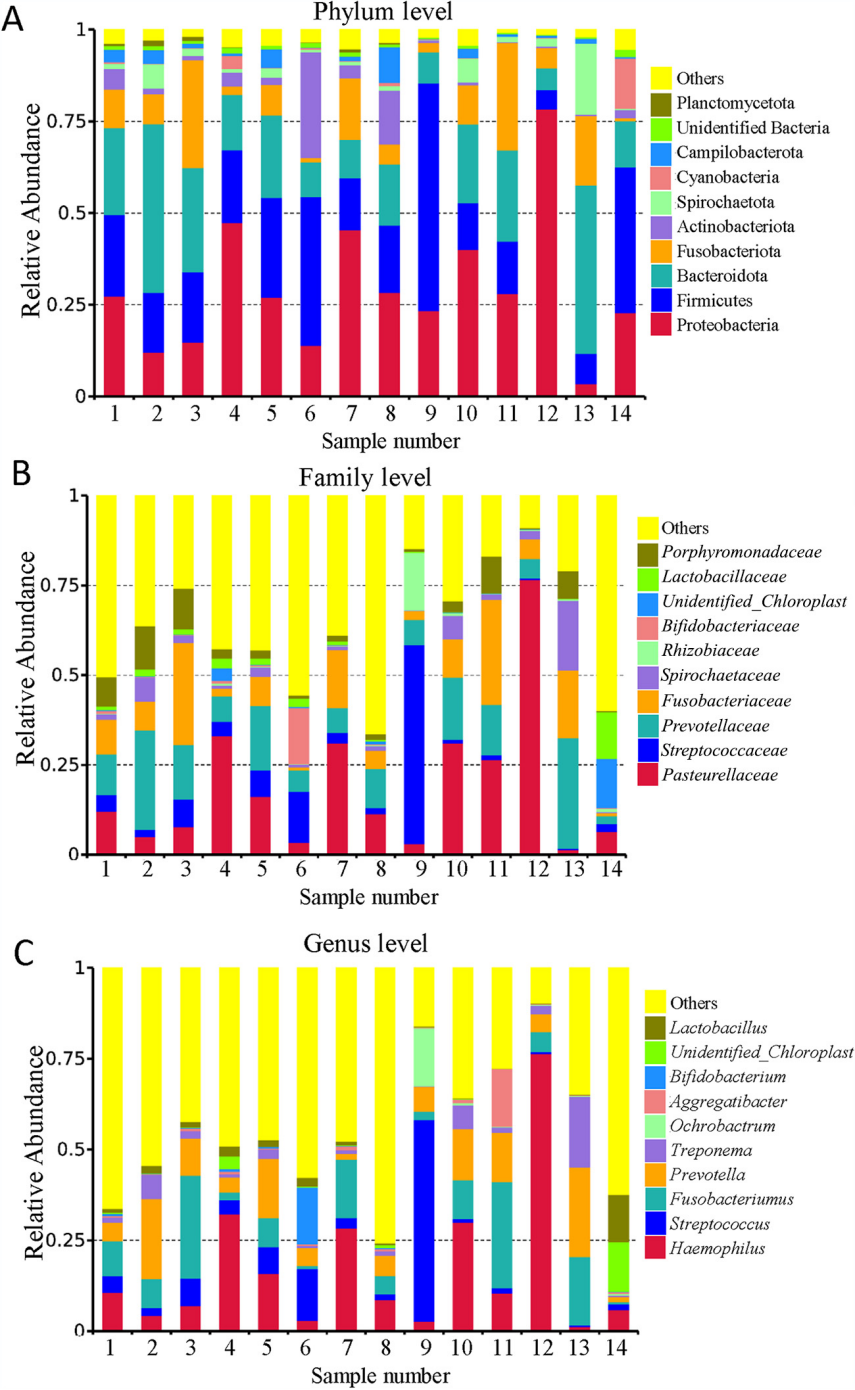
Microbiota analysis in tonsil samples using 16S rRNA sequencing. Relative abundance of bacteria at the levels of phylum (A), family (B), and genus (C) for 14 tonsillectomy samples.

To investigate the interplay between tonsil microbiota and virus infection, we selected 6 HAdV-negative samples and 8 HAdV-positive samples for microbiome analysis by bacterial 16S rRNA gene sequencing. Taxonomic profiling suggested that the human tonsil microbiota structure was dominated mainly by the Proteobacteria, Firmicutes, Fusobacteriota, and Bacteroidota phyla (Fig. 2A), which comprised almost 70% of the total phyla in the samples. This observation is consistent with previous reports that the tonsil microbiota mainly consists of these phyla (30). We further analyzed the dominant families and found that *Pasteurellaceae*, *Streptococcaceae*, *Prevotellaceae*, and *Fusobacteriaceae* accounted for more than 43% of the families (Fig. 2B). We also identified Haemophilus influenzae, Streptococcus, *Fusobacterium*, *Prevotella*, Treponema, and *Ochrobactrum* as the most prevalent genera (Fig. 2C).

### Correlation analysis of microbiota with HAdV status of tonsillectomy samples.

**FIG 3:**
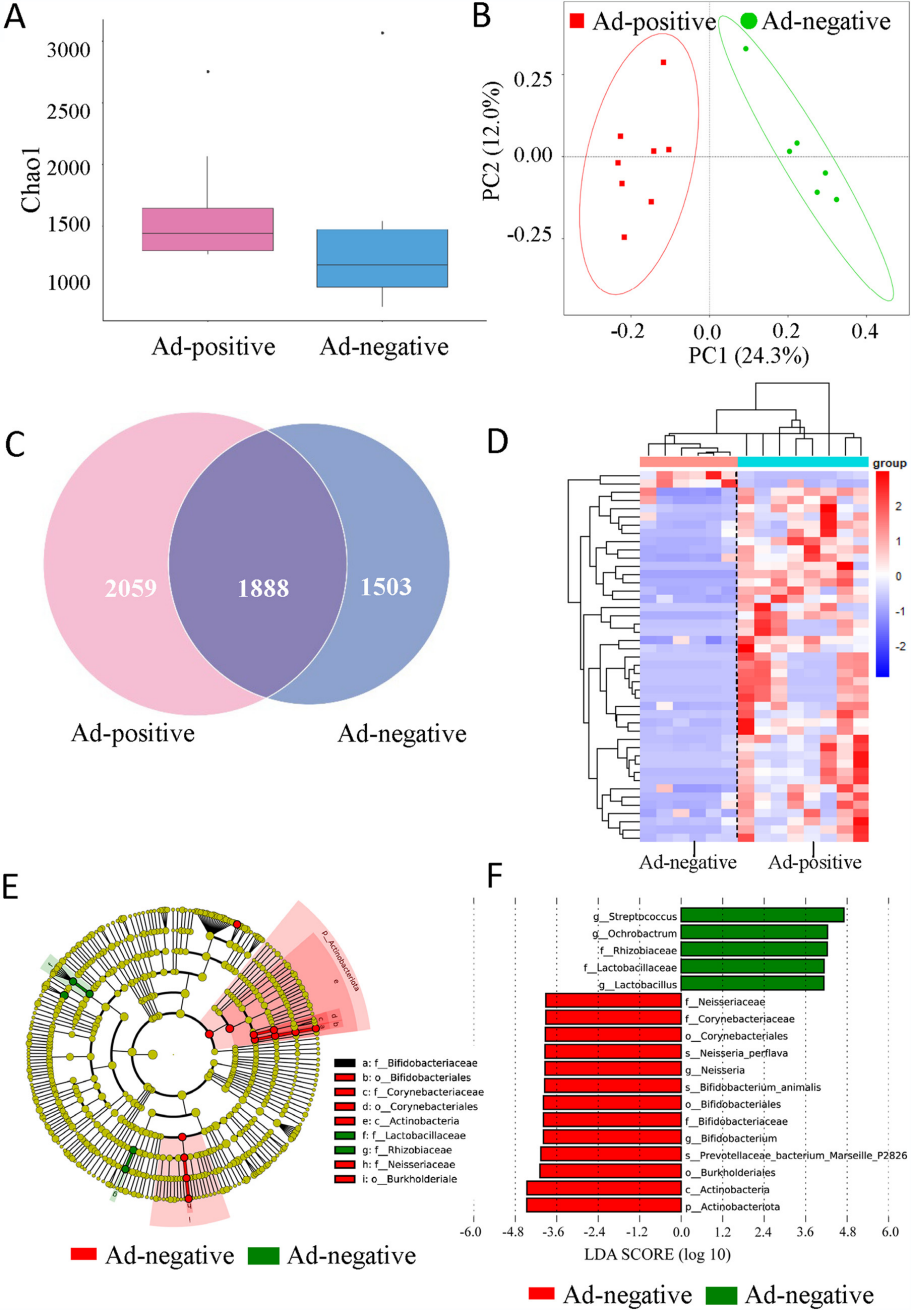
Correlation analysis of microbiota between HAdV-positive and HAdV-negative tonsils. (A) Total number of bacterial species in HAdV-positive and HAdV-negative samples determined by Chao 1. (B) Cluster separation between HAdV-positive and HAdV-negative samples using PCoA plot. (C) Venn diagram; the number of operational taxonomic units (OTUs) in HAdV-positive and HAdV-negative samples are shown. (D) Heatmap of the OTU of HAdV-positive and HAdV-negative samples. (E and F) LEfSe analysis of the discriminative microbiota between the HAdV-positive and HAdV-negative samples (linear discriminant analysis [LDA] score ≥ 3.9). HAdV-positive samples (*n* = 8) and HAdV-negative samples (*n* = 6).

For comparative analysis of the microbiota between HAdV-positive and HAdV-negative tonsil tissues, the resultant sequencing reads were processed using the QIIME software package to reveal tonsil microbiota. We estimated the total number of species used Chao 1, a widely used method for diversity analysis. The species richness was higher in the HAdV-positive group than that in the HAdV-negative group (Fig. 3A). There was also an obvious separation between HAdV-positive and HAdV-negative groups using a model of principal-coordinate analysis (PCoA) (Fig. 3B). A closer look at the microbiota community revealed by a Venn graph showed the shared and specific operational taxonomic units (OTUs) between HAdV-positive and the -negative groups (Fig. 3C). Specifically, 214 unique species were obtained after removal of those with zero or very low abundance (total abundance across samples ≤ 0.005) in all samples, and 53 of them appeared to have a significant difference in relative abundance between the two groups by *t* test statistics. To enhance the contrast, we used by-row (by bacteria)-normalized values of relative abundance to generate the heatmap (Fig. 3D; Table S1 in the supplemental material). The differences in the composition of the tonsil microbiota of the HAdV-positive and -negative groups were also explored using the linear discriminant analysis effect size (LEfSe) method. The LEfSe analysis revealed 5 and 13 significant discriminative features in the HAdV-positive and -negative groups, respectively (linear discriminant analysis [LDA] with score ≥ 3.9) (Fig. 3E). The HAdV-positive group had remarkably high abundances of Actinobacteria compared to that of the HAdV-negative group at the phylum level. The relative abundances of the *Bifidobacteriaceae*, *Neisseriaceae*, *Corynebacteriaceae*, *Lactobacillaceae*, and *Rhizobiaceae* families were potentially associated with HAdV persistency in the tonsil. Additionally, further statistical analysis demonstrated the HAdV-positive group had significantly higher abundances of *Neisseria* and *Bifidobacterium* and lower abundances of Streptococcus, *Ochrobactrum*, and *Lactobacillus* than those of the HAdV-negative group at the genera level (Fig. 3E and F). The results indicated that there was a distinct difference between the microbial communities and HAdV status in the tonsillectomy samples.

### Isolation of microbes from tonsillectomy samples.

We attempted to isolate the bacteria from the tonsil samples using different culture conditions. Among the 113 isolates randomly picked for 16S rRNA sequencing analysis, most of the isolates were of the normal flora, but some were pathogenic bacteria, including Streptococcus sp. and S. pneumoniae, Staphylococcus aureus, Capnocytophaga sputigena, Veillonella sp., *Prevotella*, Pseudomonas aeruginosa, *Neisseria*, and Moraxella catarrhalis (Table S2). It is worth noting that we have isolated relatively abundant Streptococcus, even in HAdV-positive samples. In fact, Streptococcus 16S rRNA was abundant in both HAdV-negative and -positive tonsils (Table S1), though HAdV-negative tonsils had a higher abundance than that of HAdV-positive samples (Fig. 3F). We did not compare the difference of the isolates from HAdV-positive and HAdV-negative samples since the isolates were randomly picked under nonoptimal culture conditions.

### HAdV reactivation in isolated lymphocytes by SCFAs.

**FIG 4:**
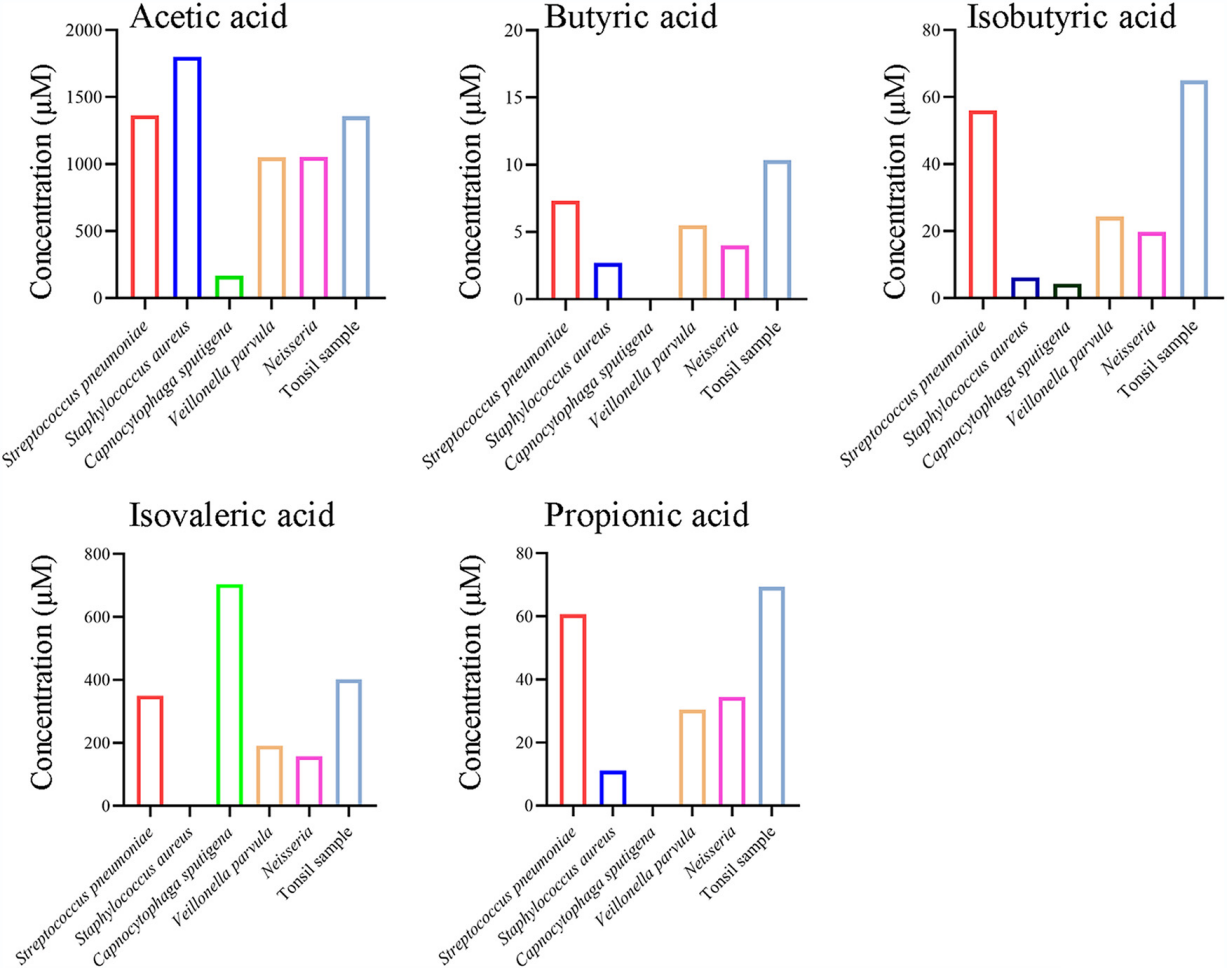
SCFAs production level of isolated bacteria and pan-bacteria in tonsil tissue. (A to E) Five representative bacteria isolated from tonsil were selected, including S. aureus, S. pneumoniae, C. sputigena, Veillonella parvula, and *Neisseria*. In addition, bacteria on tonsil samples were collected by mincing tonsils in DMEM, and the supernatant was inoculated into LB medium for 16 h cultivation and subjected for GC-MS analysis. Commercial compounds were used as standards for quantification.

We have previously shown that histone deacetylase inhibitors had the ability to promote HAdV infection (18). Microbial metabolites like SCFAs are highly produced and have demonstrated activity in histone acetylation. We asked whether bacterial metabolites had an effect on HAdV infection. We first determined SCFA production by the isolated bacterial strains as well as from minced tonsillectomy tissues. Several SCFAs, including acetic acid, butyric acid, isobutyric acid, isovaleric acid, and propionic acid, were detected from the culture medium of minced tissues or from representative microbes like S. aureus, S. pneumoniae, C. sputigena, Veillonella sp., and *Neisseria* sp. (Fig. 4). Acetic acid and isovaleric acid were among the more accumulated SCFAs in the medium of isolated bacteria and tonsil samples. Butyric acid, isobutyric acid, and propionic acid were also detected at various amounts in the medium.

**FIG 5:**
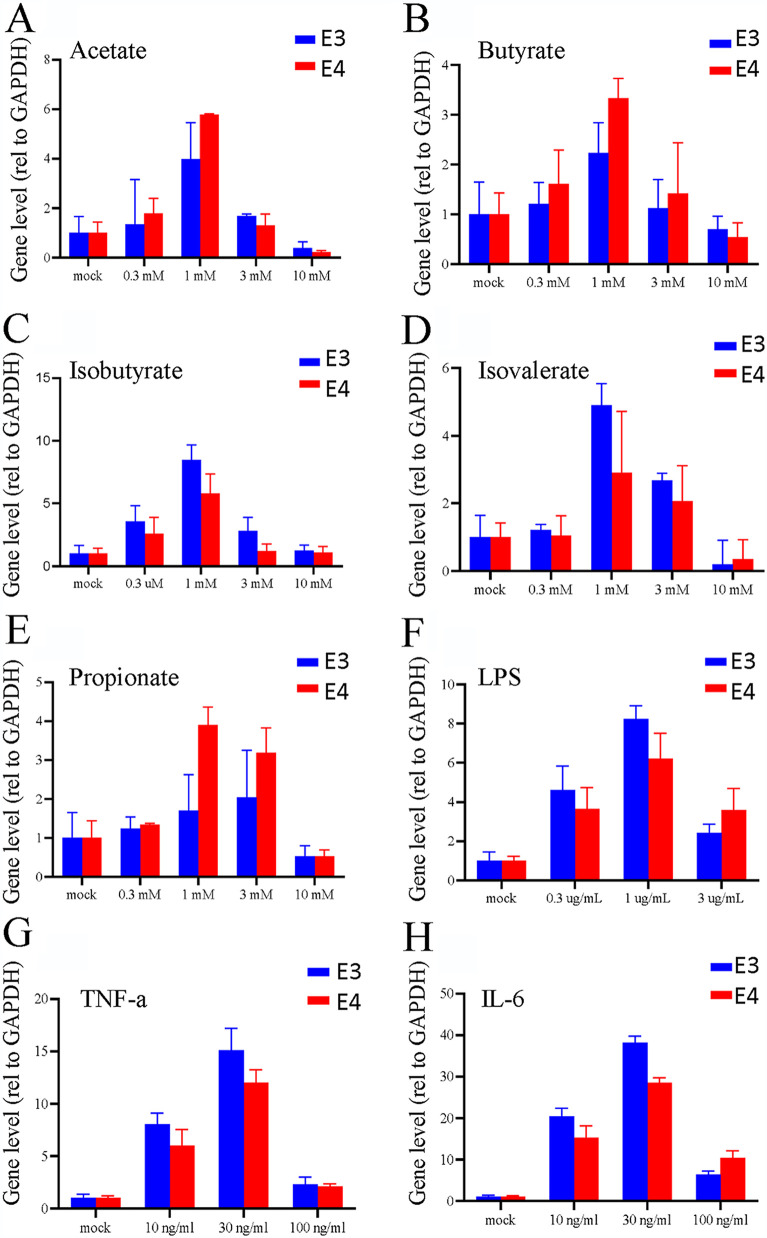
SCFAs promote adenovirus replication *in vitro*. HAdV-positive lymphocytes were isolated and cultured. The lymphocytes were seeded at 1 × 10^6^ cells/well on 12-well plates. The cells were treated with different concentrations of sodium acetate (A), sodium butyrate (B), sodium isobutyrate (C), sodium isovalerate (D), sodium propionate (E), LPS (F), TNF-α (G), and IL-6 (H) for 24 h. The supernatant was taken and treated with proteinase K to determine the levels of the HAdV early gene E3 and E4 DNA by qPCR.

We then tested the metabolites on HAdV reactivation from isolated lymphocytes. In this regard, the lymphocytes were treated with 1 mM SCFA, a noncytotoxic concentration to the isolated lymphocytes (data not shown). After treatment for 96 h, the cells and culture medium were collected and used for the detection of viral replication by quantitative PCR (qPCR) and for the recovery of infectious virions with a secondary infection. We also included LPS, a pathogen-associated molecular pattern (PAMP) of Gram-negative bacteria, and proinflammatory cytokines like tumor necrosis factor alpha (TNF-α) and interleukin 6 (IL-6) in the treatment of the lymphocytes. SCFA treatment resulted in HAdV DNA replication as was determined by HAdV E3 and E4 using qPCR (Fig. 5A to E). Similarly, we were able to recover infectious HAdV from SCFA-treated, but not from mock-treated, samples. In addition to SCFAs, we found treatment with bacterial LPS or with proinflammatory cytokines like TNF-α or IL-6 also led to increased HAdV replication (Fig. 5F to H).

### HAdV reactivation by SCFAs in a xenograft model.

**FIG 6:**
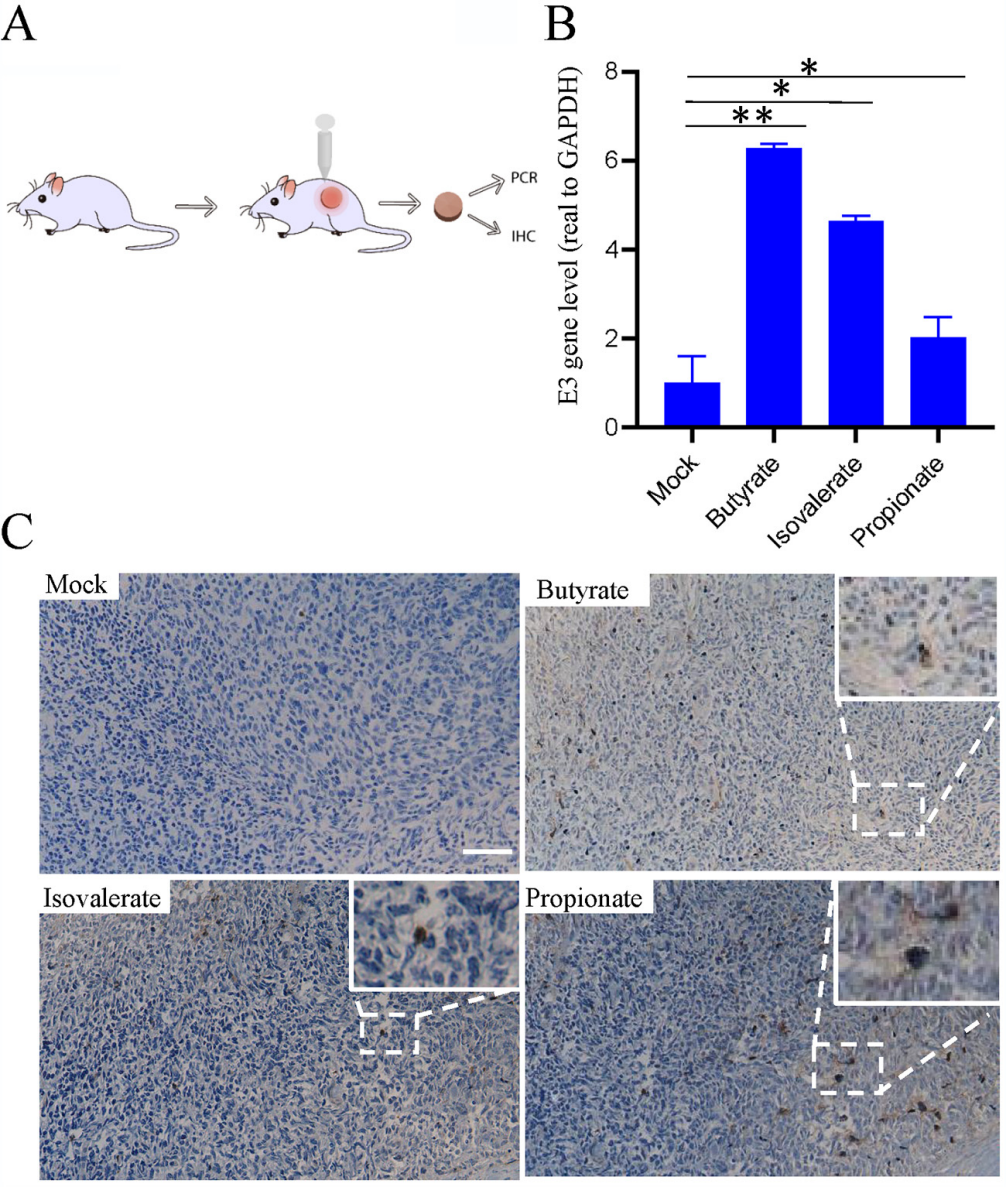
Reactivation of HAdV by SCFAs in a xenograft model. (A) Experimental design. The tonsil tissue was cut into 2 by 2 by 2-mm cubes and then implanted into the back of nude mice. The mice were treated with PBS or isobutyrate (100 mg/kg), isovalerate (100 mg/kg), and propionate (200 mg/kg) via subcutaneous injection near the tonsil transplantation spot on the 3rd, 4th, and 5th days. The tonsil tissue was taken out on the 7th day, and the presence of HAdV was detected by qPCR or immunohistochemistry (IHC). *n* = 3 for each treatment group. (B) DNA levels of HAdV early gene E3 and E4 in different treatment groups. *, *P* < 0.05; **, *P* < 0.01. (C) IHC straining of the tonsil tissue. The HAdV capsid protein, hexon, was used as the detection target. Scale bar, 25 μm.

We found that bacterial metabolites promoted HAdV reactivation in isolated lymphocytes. To extend this finding, HAdV-positive tonsil fragments were implanted in nude mice (Fig. 6A). The animals were mock treated or treated (subcutaneously [s.c.]) with sodium butyrate (100 mg/kg), isovalerate (100 mg/kg), or sodium propionate (200 mg/kg) on the 3rd, 4th, and 5th day after tonsil implantation. On the 7th day, the tonsil implants were removed for qPCR study or for immunohistochemistry (IHC) after fixation with paraformaldehyde. As shown in Fig. 6B, SCFA treatment significantly increased viral DNA replication in the xenograft model. The samples were also stained for virus protein hexon expression by IHC. As shown in Fig. 6C, there was significant expression in SCFA-treated samples, indicating SCFA treatment promoted viral replication and viral protein expression.

### SCFA treatment induces histone acetylation and acetylated histone association with viral genes or gene promoters.

**FIG 7:**
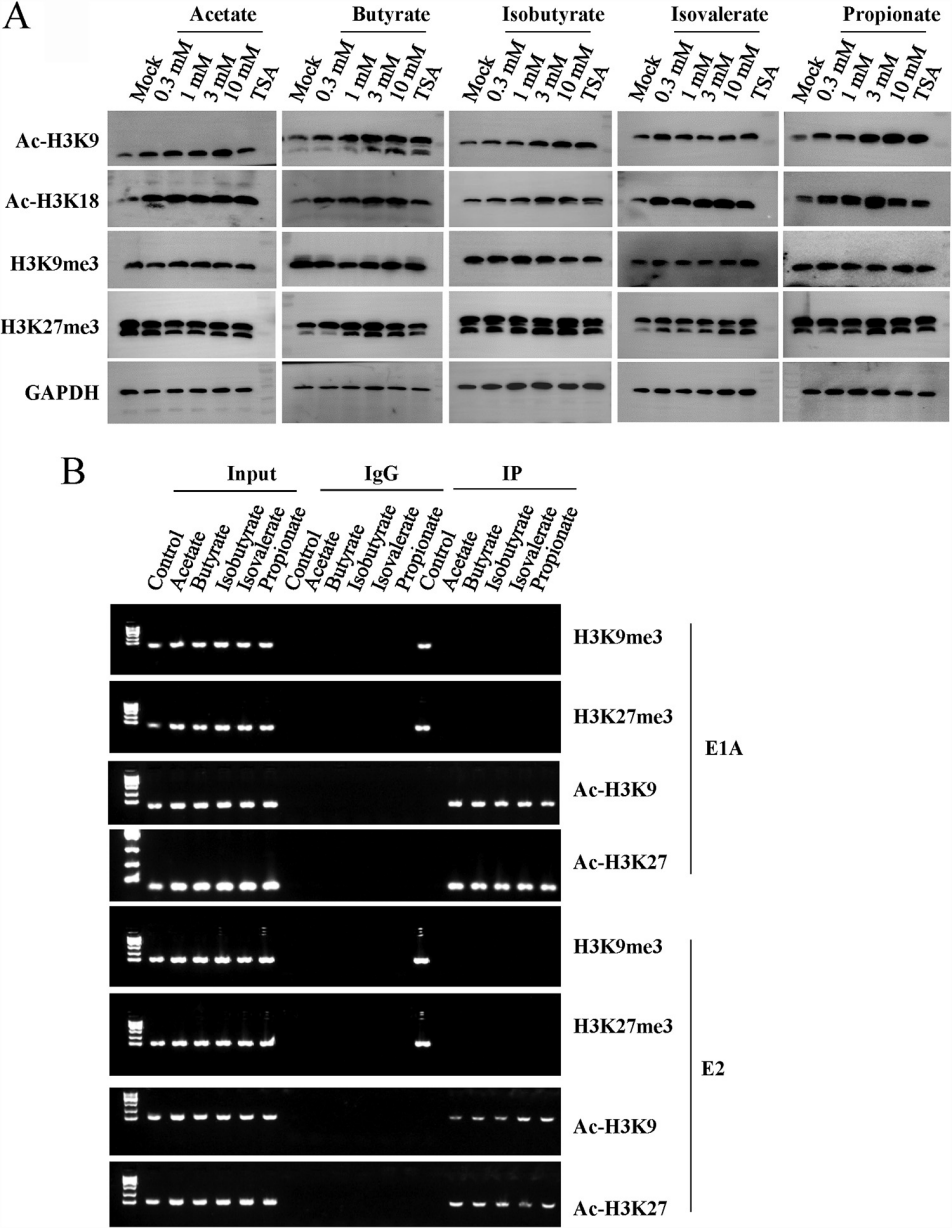
Epigenetic regulation of SCFAs on HAdV reactivation. (A) Histone modification of tonsil samples upon SCFA treatment. The HAdV-positive lymphocytes were isolated and cultured. The lymphocytes were treated with PBS or tested concentrations of SCFAs for 24 h. The protein levels of Ac-H3-K9, Ac-H3-K18, H3K9me3, H3K27me3, and GAPDH were detected by Western blot analysis. (B) Association of HAdV DNA with modified histones by ChIP assay. The lymphocytes containing HAdV were isolated and cultured. The lymphocytes were treated with PBS or tested concentrations of SCFAs for 6 h. The immunocomplexes of anti-Ac-H3-K9, Ac-H3-K18, H3K9me3, and H3K27me3 were detected for the promoter DNA of HAdV E1A and E2.

SCFAs are general inhibitors of class I and class II histone deacetylases. To investigate whether SCFA promoted HAdV reactivation through histone acetylation, isolated lymphocytes from HAdV-positive samples were treated with 1 mM acetate, butyrate, isobutyrate, isovalerate, and propionate for 24 h. Histone modifications were determined by immunoblotting studies. As shown in Fig. 7A, treatment with acetate, butyrate, isobutyrate, isovalerate, and propionate increased histone-3 (H3) acetylation (Ac-H3K9 and Ac-H3K18). In contrast, the treatment showed no significant effect on H3 methylation (H3K9me3 or H3K27me3). Treatment with trichostatin A (TSA), a well-characterized histone deacetylase (HDAC) inhibitor, increased H3 acetylation level. H3 methylation remained unchanged in the samples (Fig. 7A).

During the cycle of HAdV infection, the viral DNA is modified by histone proteins. Therefore, the effect of SCFA on HAdV reactivation was assessed by chromatin immunoprecipitation (ChIP) assay for histone-3 association with viral early gene or gene promoters. At treatment with SCFA samples, we detected significant increases of viral DNA of the E1A and E2 promoter regions in the anti-Ac-H3K9 and anti-Ac-H3K18 immunocomplexes (Fig. 7B), while no E1A or the E2 region DNA was detected in the anti-H3K9me3 or the anti-H3K27me3 immunocomplexes. On the contrary, without treatment as a control, we detected viral DNA of the E1A and E2 promoter regions in the anti-H3K9me3 and the anti-H3K27me3 immunocomplexes, while no E1A or the E2 regions were detected in the anti-Ac-H3K9 and anti-Ac-H3K18 immunocomplexes. The results implied that SCFAs produced by tonsil microbiota may serve as trigger compounds to promote HAdV reactivation in tonsil samples.

In summary, we found there was a significant difference in the microbiota on HAdV-positive and HAdV-negative tonsil samples. Although the effect of HAdV on the microbiota remains to be investigated, this study shows that bacterial products like LPS and metabolites like SCFAs have the potential to induce HAdV reactivation, linking the bacterial microbiome to HAdV reactivation, which may lead to recurrent viral infection.

## DISCUSSION

Adenovirus infections continue to be a major problem among the general pediatric population, immunocompromised patients, and HSCT recipients (15, 31, 32). Following primary infection, the virus is able to establish latent/persistent infection majorly residing in tonsils. Inflammation of the tonsils with subsequent obstructive hypertrophy is one of the most ancient and common pediatric problems. We and others have reported high prevalence of latent HAdV in tonsillectomy samples (17, 18, 20, 33). Reactivation of adenovirus from latency or persistency has been suspected as a primary source of recurrent tonsilitis, even though little attention has been paid to the mechanism by which HAdV reactivation is regulated.

As a natural reservoir of viruses, tonsils are also subjected to many other pathogenic microorganisms, including both virus and bacteria (34, 35). Given that tonsils are located at the gateway of the respiratory tracts and are commonly exposed to bacterial pathogens, we questioned whether tonsil microbiota play a role in viral reactivation and impact on recurrent disease. With this purpose, we first studied the microbiota of tonsillectomy samples. We identified a broad spectrum of bacteria in the samples by 16S rRNA gene sequencing and bacterial culturing. Interestingly, a differential correlation in HAdV status to that of tonsil microbiota was revealed. The HAdV-positive group has significantly higher abundances of *Neisseria* and *Bifidobacterium* and lower abundances of Streptococcus, *Ochrobactrum*, and *Lactobacillus* than the HAdV-negative group at the genera level. These observations suggested a possible interplay between bacteria and virus, two types of microbial residents on tonsils.

HDAC inhibitors and reagents promoting PKC signaling cause HAdV reactivation (18, 25), while microbiota metabolites, including the SCFAs, which are known to exhibit activity on histone acetylation (27, 36, 37). We thus questioned whether there was an interplay between bacterial colonization and HAdV reactivation through bacterial metabolites. With this purpose, we investigated the impact of bacterial metabolites on HAdV reactivation on tonsil tissue. We characterized metabolites from culture medium of minced tissues or from isolated microbes and detected the presence of SCFAs with various amounts. Those compounds were further confirmed to enable HAdV reactivation on tonsillectomy species. In addition, proinflammatory stimuli such as LPS, TNF-α, and IL-6 also promoted histone acetylation and induced HAdV DNA replication in treated tonsil lymphocytes. The latency or complete silencing of HAdV genome undoubtedly involves multiple epigenetic silencing mechanisms, including DNA methylation and specific patterns of histone methylation (22, 38, 39). In order to assess the effect of SCFAs on HAdV reactivation, we first investigated whether these molecules also impact histone acetylation and methylation. We found that SCFAs increased the level of histone acetylation with little change at the methylation level. Enrichment of viral DNA of the E1A and E2 promoter regions in the anti-Ac-H3K9 and anti-Ac-H3K18 immunocomplexes was observed, with decreased amount in the anti-H3K9me3 and the anti-H3K27me3 immunocomplexes.

We found that treatment with SCFAs promoted viral DNA amplification by a 4- to 8-fold increase in primary lymphocytes. Similar levels of increases were observed using commercial HDAC inhibitors (18), suggesting that epigenetic factors regulate HAdV reactivation. The mechanism by which HAdV reactivates has to be determined. Garnett and colleagues reported that PMA and ionomycin stimulation of HAdV-harboring primary lymphocytes for 44 h caused reactivation of HAdV. Viral DNA amplification seemed to be at relatively low levels in those cells since a round of further amplification in the susceptible A549 cells for 10 days resulted in viral DNA changes by 2 to 3 magnitudes for most of the samples (20). Nonetheless, these studies underline the importance of virus latency in recurrent infection.

The interplays between bacteria and viruses play a fundamental role in health and diseases (40, 41). Microbiota can facilitate virus replication by many other mechanisms. Microbiota could stabilize virions, which potentially enhances viral transmissibility, and promote virus attachment to host cells. Bacterial surface polysaccharides could promote virion stability and prevent premature conformational changes that result in genome extrusion into target cells on receptor binding. Virus bound by bacterial outer membrane component LPS and other *N*-acetylglucosamine-containing bacterial surface polysaccharides had enhanced infectivity (42). Virus isolated from microbially colonized mice had higher viral infectivity than that from the lumens of antibiotic-treated or germ-free mice (43).

Microbiota could also regulate host immune response to virus infection. Microbiota hinders the activation of antiviral humoral responses, mainly through regulating the production of virus-specific antibodies (44). Microbiota is not only for the eliciting of effector immune responses by stimulating the production of various proinflammatory cytokines such as interferon gamma (IFN-γ) during infection but also for the establishment of an immunotolerant microenvironment by contributing to the generation of immunoregulatory cells such as regulatory T (Treg) cells to maintain homeostasis (45–47). This suggests that microbiota-induced Treg cells and Treg cell-related cytokines limit the degree of antiviral immune responses.

In turn, the invading viruses and commensal microbiota could have suppressive outcomes for viral infection. Microbiota help maintain robust antiviral immunity, which essentially guarantees effective elimination of invading viruses. For instance, microbiota modulates type I interferon and antibody-mediated immune responses in chickens against influenza virus infection (48). Viruses are exposed to mucosal surfaces (e.g., vaginal, respiratory, or gastrointestinal tract [GI]), and they have three broad lines of defense to overcome, including the mucus layer, innate immune defenses, and adaptive immune defenses (49). The microbiota enhances mucosal barrier function and secretion of antiviral antimicrobial peptides (AMPs) or bacteriocins (50–52).

The microbiome is highly dynamic during this period of life due to the influence of several host-related and environmental factors, including virus infection and antibiotic exposure. In this study, a differential correlation in HAdV status to that of tonsil microbiota was revealed. This observation might also suggest viral status could impact the microbiota. Respiratory viral infection induces microbiome alteration and predisposes patients to secondary bacterial infections, which can have a more severe clinical course (53). Acute otitis medium is commonly caused by bacterial pathogens. It was reported that children infected with adenovirus are more likely to develop acute otitis media (54, 55). Viral infection in general increases glucose uptake and the fermentation process, complicating bacterial colonization and upper respiratory tract infection (56, 57). All these observations indicate specific viral infections have been shown to increase the chance and virulence of bacterial infections. In turn, the substantial perturbations in the microbiota resulted from virus infection could cause dysbiosis in the host, which may further affect the viral infectivity.

In summary, we uncovered a differential correlation of microbiota and HAdV status in tonsillar samples. We showed that bacterial metabolites promote HAdV reactivation. Cross talk between bacteria and virus possibly leads to a more successful colonization and virus persistence. Recurrent virus infection might be related to the change of microbiome in patients. Understanding the detailed mechanism of bacteria-virus interplay would thus provide fundamental instructions for dealing with clinical complications of bacteria-virus coinfection as well as recurrent tonsilitis.

## MATERIALS AND METHODS

### Tonsil samples.

Research was approved by the Ethics Committees of Nanjing Medical University and Medical School of Nanjing University, and informed consent was obtained from legal guardians involved in this study in accordance with the Declaration of Helsinki. Tonsil samples from 81 patients who underwent tonsillectomy with a history of recurrent tonsillitis between September 2018 and September 2020 were collected. Fresh tonsil samples from patients undergoing routine tonsillectomies for chronic tonsillitis or hypertrophic tonsils were collected at Nanjing Children’s Hospital. At the time of surgery, none displayed symptoms of acute respiratory infection. All children had clinical symptoms due to hypertrophy of adenoids. None of the patients had symptoms that would indicate acute adenovirus infection. The tissue samples were processed within 4 h after surgeries. After being thoroughly rinsed with ice-cold Dulbecco’s modified Eagle medium (DMEM) with or without antibiotics, the samples were used immediately for lymphocyte isolation or culturing or stored away at −80°C after being snap-frozen for further studies.

### Reagents and antibodies.

Sodium acetate (catalog no. S2889), sodium butyrate (catalog no. 303410), sodium propionate (catalog no. P5436), sodium salt of isobutyric acid (catalog no. I1754), and isovaleric acid (catalog no. 129542) were purchased from Sigma-Aldrich. The compounds were used in the form of sodium salt. Antibodies to acetyl-H3-K9 (Abclonal; catalog no. A7255), acetyl-H3-K18 (Abclonal; catalog no. A7257), trimethyl-H3-K9 (Beyotime; catalog no. AF5707), trimethyl-H3-K27 (Beyotime; catalog no. AF5710), and GAPDH (glyceraldehyde-3-phosphate dehydrogenase) (Abcam; catalog no. AB8245) were obtained commercially. PrimeScript RT master mix (TaKaRa; catalog no. RR047A) and SYBR green PCR master mix (catalog no. Q141-02/03; Vazyme, Nanjing, China) were obtained commercially. Lymphocyte separation reagent was purchased from Tianjin Haoyang TBD (catalog no. LTS1077). EasySep human CD3^+^ selection cocktail II (Stem Cell; catalog no. 17851), human CD19^+^ selection cocktail II (Stem Cell; catalog no. 17854), CD2-APC (BioLegend; catalog no. 300311), and CD19-PE (BioLegend; catalog no. 302207) were purchased from commercial sources. Horseradish peroxidase (HRP)-conjugated secondary antibodies were purchased from Sigma-Aldrich. Immunoblots were detected using an ECL reagent kit (Beyotime) and the ChemiScope 6000 Touch imaging system (Clinx, China).

### Bacterial 16S rRNA gene sequencing.

DNA was isolated from snap-frozen tonsil tissues and used for 16S rRNA gene amplification by PCR using a universal primer pair of 515F (GTGCCAGCMGCCGCGGTAA) and 806R (GGACTACHVGGGTWTCTAAT). The primer pair anneals to the V4 region of the bacterial 16S rRNA gene. PCR products were purified with GeneJet gel extraction kit (Thermo Scientific) for the construction of sequencing libraries using Illumina TruSeq DNA PCR-free library preparation kit (Illumina, USA) by following the manufacturer’s recommendations. Trimmomatic software was used for quality control and FLASH (Fast Length Adjustment of SHort reads) software was used for splicing. The taxonomic unit was screened for further annotation. PCoA plots were generated by QIIME script (make_2d_plots.py). Heatmap was generated based on normalized bacteria abundance using an R function, heatmap (a function in R stats) (58). To find out the relative abundance differences between the HAdV-positive and HAdV-negative groups, the sequences were selected after removal of those with zero or very low abundance (total abundance across samples ≤ 0.005) in all samples and used for the analysis. To enhance the contrast, we used by-row (by bacteria)-normalized values of relative abundance to generate the heatmap.

### Isolation of tonsil bacteria with agar plates.

Freshly removed tonsil specimens were rinsed twice in a 50-ml conical Falcon tube with ice-cold DMEM without antibiotics. The samples were minced in 1 ml DMEM into fine pieces using razor blades. The samples were then centrifuged at 1,000 rpm for 5 min to release bacteria from tonsil pieces. The supernatants were collected and then serially diluted for the recovery of culturable organisms on blood or chocolate agar plates. After incubation at 37°C aerobically or anaerobically for 36 to 48 h, individual colonies were collected and subcultured into the same medium from which they were isolated under an appropriate atmosphere. The isolates were identified by 16S rRNA analysis using the universal primers 27F (5′-GAGAGTTTGATYMTGGCTCAG) and 1492R (5′-TACGGYTACCTTGTTACGACTT). A glycerol stock of each isolate was stored at −80°C.

### Sample preparation and metabolomics.

The metabolites of bacteria were quantified using gas chromatography-mass spectrometry (GC-MS) (Trace 1310-TSQ8000 Evo; Thermo, USA) following previous protocols (59). A stock solution of a bacterium was cultured in 2 ml liquid culture at 37°C with agitation overnight. A 100-μl seed culture was transferred to 15 ml fresh LB medium and cultured for 16 h. The metabolites in the supernatants were analyzed by GC-MS. Commercially obtained acetate, butyrate, isobutyrate, isovalerate, and propionate were used as standards. We used a TG-5MS capillary GC column (0.25 mm x 30 m, 0.25 mm; Thermo Fisher), using split ratio of 20:1 with 50°C for 0 to 2 min, 50°C to 70°C for 2 to 4 min, 70°C to 85°C for 4 to 9 min, 85°C to 110°C for 9 to 14 min, 110°C to 290°C for 14 to 20 min, and 290°C for 20 to 28 min to separate the substances. A 70-eV electron ionization (EI) source was adopted, with 290°C ion transmission line, 230°C ion source, and 1.2 ml/min high-purity helium used as the carrier gas, under full-scan mode. The scan range was 30 to 600 *m/z* to detect and analyze substances.

### Lymphocyte preparation.

To isolate lymphocytes, freshly removed tonsil samples were rinsed with ice-cold RPMI 1640 medium containing penicillin-streptomycin and amphotericin B and were minced into single cells using a razor blade. After passing through a Falcon cell strainer, the cells were collected by washing twice with RPMI 1640 and used for cell population selection or for cell culture studies.

We used the EasySep kit to separate the lymphocytes into CD3^+^ and CD19^+^ cells by following the manufacturer's instructions. Cells were eluted from the magnetic beads by adding cell-specific antibody to compete with bead-coupled antibody. To determine the purity of the cell populations after magnetic bead separation, the cells were stained with CD2-APC for CD3^+^ T cells and CD19-PE for CD19^+^ B cells and analyzed on a BD FACSCalibur instrument installed with CellQuest software. In general, a 95% or higher purity was achieved after bead separation.

### *In vitro* reactivation study.

To test conditions for HAdV reactivation, freshly isolated tonsil lymphocytes resuspended in complete RPMI 1640 medium were plated in 24-well plates at 1 × 10^6^ cells/well. The cells were treated with sodium acetate, butyrate, isobutyrate, isovalerate, or propionate (0.3 mM to 10 mM) and cultured at 37°C for 96 h. At the end, the cells and the culture medium were collected. After freeze-thaw 3 times in liquid nitrogen and a 37°C water bath, the samples were used either for PCR studies or secondary infection assays.

### Xenograft model and *in vivo* activation studies.

We used a xenograft model to investigate HAdV reactivation *in vivo* as described previously (18). All experimental procedures were carried out strictly in accordance with the guide for the care and use of laboratory animals and the related ethical regulations instilled at the Medical School of Nanjing University. Female BALB/c athymic mice (catalog no. D000521) of 4 to 6 weeks of age were purchased from Model Animal Research Center of Nanjing University and were housed in microisolator cages under specific-pathogen-free conditions on a 12:12 h light/dark cycle with free access to food and water. The tonsils were thoroughly washed and then cut into small cubes of approximately 2 mm in each dimension. Tissue fragments were surgically implanted in the rear flanks (one on each side) of mice under anesthesia. The surgery procedure resulted in minimal postoperative morbidity and mortality. On day 3 after the surgery, mice (3 per group) were randomly grouped and treated with a vehicle (saline, 50 μl), sodium butyrate (100 mg/kg), sodium isovalerate (100 mg/kg), or sodium propionate (200 mg/kg) by subcutaneous injection (s.c.) near the sites of the implants for 3 consecutive days. The tissues were removed on day 7 and were tested for detection of viral DNA replication by qPCR.

### DNA extraction.

Freshly removed tonsil tissues (approximately 20 mg) or isolated tonsil lymphocytes (1 × 10^7^) were lysed in 100 μl buffer containing 150 mM NaCl, 0.5% NP-40, 0.1% SDS, and 20 mM Tris-HCl (pH 7.6). The lysate was then treated with proteinase K (0.2 mg/ml) at 55°C overnight. After inactivation of proteinase K at 95°C for 10 min, the DNA was purified by phenol-chloroform extraction and ethanol precipitation. Total DNA was resuspended in Tris-EDTA (TE) buffer, and the concentration was determined using a NanoDrop 2000 spectrometer.

### PCR and real-time PCR.

Typically, the PCR was carried out in 25 μl volume using *Taq* DNA polymerase (Vazyme, China; catalog no. P211-01) and 1 μg total DNA for amplification. PCR amplification started with denaturation for 3 min at 94°C, followed by 30 cycles of 30 s at 94°C, 30 s at 58°C, and 30 s at 72°C, followed by a final extension at 72°C for 10 min. The products were analyzed by electrophoresis and automated DNA sequencing analysis. The DNA products or cDNA was examined by PCR using primers that anneal to the E3 and E4 early gene, a conserved region of the species C or B adenovirus by RT-qPCR with a 7300 real-time PCR system (Thermo Fisher). Cellular GAPDH was used for normalization. The data were analyzed using the threshold cycle (2^−ΔΔ^*^CT^*) method for relative quantification following the MIQE guidelines (60). The primer sequences are listed in Table 3.

**TABLE 3:** List of primers used for HAdV, EBV, and HCMV characterization and ChIP assays

Primer name	Sequence (5′–3′)	Size (bp)	Position (nucleotide range)	GenBank accession no.
HCMVF	CAGTCCGTCCGTCCAAAGAA	959	82170–83128	NC_006273
HCMVR	CAACCAAACCAGCGTCAAGG	959	82170–83128	NC_006273
HexF1	AACTTCCAGCCCATGAGC	435	21274–21708	J01917.1
HexR1	CAGGTACACGGTCTCGATGA	435	21274–21708	J01917.1
HexF2	GACAGCTATGATCCAGATGT	1,245	20047–21291	J01917.1
HexR2	GCTCATGGGCTGGAAGTT	1,245	20047–21291	J01917.1
YWHexF	GTTGACAGCATTACCCAGA	265	21407–21671	AC_000008.1
YWHexR	GCGTGCGCAGGTACA	265	21407–21671	AC_000008.1
EBNA1F	AGGCTGCCCACCCTGAGGAT	170	35882–36051	NC_007605
EBNA1R	GCCACCTGGCAGCCCTAAAG	170	35882–36051	NC_007605
E1AF	TGCAAGTGTGGCGGAACA	159	126–284	AC_000008.1
E1AR	TCCCGCGAAAATGGCCAA	159	126–284	AC_000008.1
E2F	TGCAAGCCATCAACAAAGCC	244	26048–26291	AC_000008.1
E2R	CCTCCTCCTCGTCCAAAACC	244	26048–26291	AC_000008.1
E3F	ACCACTGCTACCGGACTAAC	90	29510–29599	J01917.1
E3R	AAACCACCACATGTCCAAGC	90	29510–29599	J01917.1
E4F	CGGTAAACACATCAGGTTGGT	100	35304–35403	J01917.1
E4R	GCTGTAATGTTGTCTCTACGCC	100	35304–35403	J01917.1

### FISH.

A hexon FISH probe was generated by PCR amplification using genomic DNA of HAdV-positive tonsil sample as the template. Biotin was conjugated to the probe according to biotin random primer DNA labeling kit (Beyotime; catalog no. D3118). FISH was performed in tonsil tissues according to the manufacturer’s instructions. In brief, a 5-μm-thick section was deparaffinized in xylene and rehydrated through graded alcohols. The slides were then immersed in proteinase K (20 μg/ml) at 37°C for 20 min and blocked with the Biotin-blocking solution (Beyotime; catalog no. P0101). The hexon probe was heated at 95°C for 5 min and immediately cooled in ice. The slides were pretreated with hybrid fluid at 55°C for 30 min, followed by the addition of the hexon probes and incubated at 55°C overnight. The hybrid fluid was discarded on the second day. After the slide washing in 2× SSC (1× SSC is 0.15 M NaCl plus 0.015 M sodium citrate) solution, the slide was stained with the 3,3-diaminobenzidine (DAB) detection kit (Beyotime; catalog no. P0202). Samples were analyzed using Olympus D53.

### Immunohistochemistry.

Tissue was sectioned (5 μm) and stained using immunohistochemical techniques according to the manufacturer's instructions. In brief, samples underwent deparaffinization in an ethanol gradient, followed by washing in phosphate-buffered saline (PBS). Endogenous peroxidase activity was treated with 3% hydrogen peroxide for 10 min at room temperature. After blocking the sections with 5% bovine serum albumin (BSA), the sections were incubated at 4°C with rabbit anti-hexon (1:500) antibody (ABmart, Shanghai China) overnight, followed by HRP-conjugated secondary antibody for 1 h. The sections were developed by incubation with DAB (Beyotime) substrate.

### ChIP assay.

The ChIP assay was carried out to determine viral DNA interactions with histone proteins. Briefly, the isolated lymphocyte cells (5 × 10^7^) in 10-cm dishes were untreated or treated with acetate (1 mM), butyrate (1 mM), isobutyrate (1 mM), isovalerate (1 mM), or propionate (1 mM) for 6 h. The chromatin suspensions were prepared using a ChIP assay kit from Beyotime by following the manufacturer's instructions. Antibodies against Ac-H3K9, Ac-H3K18, and H3K27me3 or H3K9me3 were used for immunoprecipitation. The end ChIP products were isolated using a DNA purification kit (Beyotime), and the DNA was used for PCR amplification using primers targeting the vicinity of the HAdV early gene promoters or the nearby regions. The primers are listed in Table 3.

### Ethics statement.

A human subject protocol for the use of surgically removed tonsil samples was approved by Nanjing Children's Hospital. An animal care and use protocol was approved by the IACUC of Nanjing University.
