# Supplementary material for: Characterization of Tonsil Microbiota and Their Effect on Adenovirus Reactivation in Tonsillectomy Samples
Source: Microbiol Spectr. 2021 Oct 20;9(2):e01246-21. doi: 10.1128/Spectrum.01246-21 (PMC8528100; doi:10.1128/Spectrum.01246-21)
Supplement: SUPPLEMENTAL FILE 2 — Supplemental material. Download SPECTRUM01246-21_Supp_2_seq11.pdf, PDF file, 0.1 MB [file spectrum01246-21_supp_2_seq11.pdf]

Table S2. List of tonsil microbes isolated on agar plates

Isolates identified by 16S rRNA
Streptococcus sp
Streptococcus sanguinis
Streptococcus salivarius
Streptococcus infantis
Streptococcus pneumoniae
Streptococcus orail
Staphylococcus aureus
Staphylococcus sp
Staphylococcus intermedius
Capnocytophaga sputigena
Veillonella sp.
Veillonella parvula
Prevotella buccalis
Prevotella nanceiensis
Alloprevotella rava
Selenomonas infelix
Pseudomonas aeruginosa
Schaalia odontolytica
Peptostreptococcus stomatis
Weissella
Neisseria
Pediococcus pentosaceus
Selenomonas sputigena
Moraxella catarrhalis
Kingella denitrificans
Rothia mucilaginosa
unclassified bacteria

Table legend for SI tables

Table S1. 16S rRNA sequencing results of the microbiota in HAdV-negative and HAdV-positive tonsil samples. Total DNA extracted from tonsil samples was used as template for bacterial 16S rRNA gene amplification followed by sequencing library construction. After sequencing and removal of those with zero or very low abundance (total abundance across samples ≤ 0.005) in all samples, the relative abundancy differences between the HAdV-positive and HAdV-negative groups were shown in this table. These values were used to generate a R function heatmap as shown in Fig. 3D. *, $p<0.05$, **, $p<0.01$, ***, $p<0.001$.

Table S2. List of tonsil microbes isolated on agar plates. Bacteria from freshly removed tonsil specimens were isolated by agar plates cultured under aerobic or anerobic conditions. Individual colonies were collected and identified by 16S rRNA analysis.
